# From Anatomy to Outcome: Linking Glioma Location Patterns to Survival Using Non-Negative Matrix Factorization

**DOI:** 10.1007/s00062-026-01642-8

**Published:** 2026-03-27

**Authors:** Marianne Schell, Joel Kohler, Martha Folty-Dumitru, Haidar Alzaid, Irada Pflüger, Katharina Schregel, Tim Hilgenfeld, Julius Kernbach, Sandro Krieg, Felix Sahm, Wolfgang Wick, Martin Bendszus, Jessica Jesser

**Affiliations:** 1 Neuroradiology, University Hospital Heidelberg, Heidelberg, Germany; 2 Division for Computational Neuroimaging, University Hospital Heidelberg, Heidelberg, Germany; 3 Neuroradiology, University Hospital Bonn, Bonn, Germany; 4 Division for Computational Radiology & Clinical AI, University Hospital Bonn, Bonn, Germany; 5 Neurosurgery, University Hospital Heidelberg, Heidelberg, Germany; 6 Neuropathology, University Hospital Heidelberg, Heidelberg, Germany; 7 German Cancer Research Center, Heidelberg, Germany; 8 Neurology, University Hospital Heidelberg, Heidelberg, Germany

**Keywords:** Brain, Glioma, Survival prediction, Imaging biomarkers, Tumor location, Cox regression

## Abstract

**Purpose:**

The anatomical distribution of IDH-wildtype gliomas may encode prognostic information not captured by conventional clinical or molecular markers. The purpose of this study was to determine whether continuous, data-driven spatial patterns of tumor involvement provide prognostic value beyond established clinical and molecular factors.

**Methods:**

We applied non-negative matrix factorization (NMF) to preoperative tumor segmentations from 429 patients with IDH-wildtype gliomas to identify coherent spatial components of tumor involvement. Six reproducible spatial signatures were extracted and integrated into multivariate Cox proportional hazards models. Models additionally included tumor volume, age, sex, O6-methylguanine-DNA methyltransferase (MGMT) promotor methylation status, Eastern Cooperative Oncology Group (ECOG) performance status, and extent of resection.

**Results:**

All six spatial patterns demonstrated significant association with overall survival (*p* ≤ 0.005). Notably, involvement of the left parietal-occipital regions was linked to the poorest outcomes, while temporal patterns showed weaker associations with survival. Incorporating spatial patterns into the prognostic model improved its discriminatory power and altered the influence of tumor volume, indicating an interaction between tumor location and tumor burden.

**Conclusion:**

Continuous spatial phenotyping of IDH-wildtype gliomas using NMF captures prognostically relevant information not reflected by conventional markers. Integration of spatial patterns into prognostic models enhances risk stratification and supports the incorporation of detailed spatial analyses into radiologic workflows.

**Supplementary Information:**

The online version of this article (https://doi.org/10.1007/s00062-026-01642-8) contains supplementary material, which is available to authorized users.

## Introduction

Radiological assessment of IDH-wt (isocitrate dehydrogenase wildtype) gliomas often fails to capture the spatial complexity and prognostic implications of tumor behavior. Current classification systems, including the WHO CNS5 criteria [1], primarily rely on molecular and histopathological markers for grading and prognostication. While molecular markers, such as IDH mutation status and O6-methylguanine DNA-methyltransferase (MGMT) promoter methylation, have significantly advanced glioma classification, they provide limited information on spatial phenotypes. Gliomas exhibit substantial biological and molecular heterogeneity, complicating accurate classification and clinical prognostication [2–5]. Although molecular diagnostics have improved risk stratification, they provide limited insight into anatomical variation, resulting in an incomplete prediction of disease trajectory [6]. Furthermore, discrepancies between imaging findings and histomolecular profiles persist, reflecting the challenge of correlating radiographic appearance with the underlying tumor biology [7, 8].

Tumor location is widely recognized as a prognostic determinant, influencing clinical presentation, surgical accessibility, and overall survival [9–14]. Yet, in most routine imaging workflows, the location of gliomas is often reduced to simplistic labels, such as “frontal lobe” or “crossing midline”, which do not capture the complex and diffuse growth pattern characteristic. Recent voxel-based lesion mapping studies have started to reveal spatial trends specific to different subtypes of gliomas; however, these valuable insights are rarely incorporated into prognostic models [10, 15].

Emerging evidence suggests that gliomas, particularly IDH-wildtype, follow non-random spatial distributions influenced by intrinsic properties (e.g., cell-of-origin, transcriptional subtype) and extrinsic factors (e.g., white matter tracts, vascular networks, and proximity to neurogenic zones) [10, 16]. However, these tendencies are rarely modeled in a structured, quantitative way.

Integrating spatial data into computational models has the potential to address this unmet need. By defining tumor location as a structured imaging phenotype, radiology enhances predictive modeling by incorporating spatial data into prognostic assessment. Previous studies have demonstrated correlations between spatial imaging features and molecular tumor markers [14, 17–21].

Building on these observations, we transform anatomical tumor distribution into a structured, continuous imaging phenotype. By explicitly modeling spatial organization, we aim to enhance prognostic stratification beyond conventional descriptors and emphasize the neuroanatomical context as a relevant dimension of glioma biology.

## Results

### Patient Cohorts

**Fig. 1:**
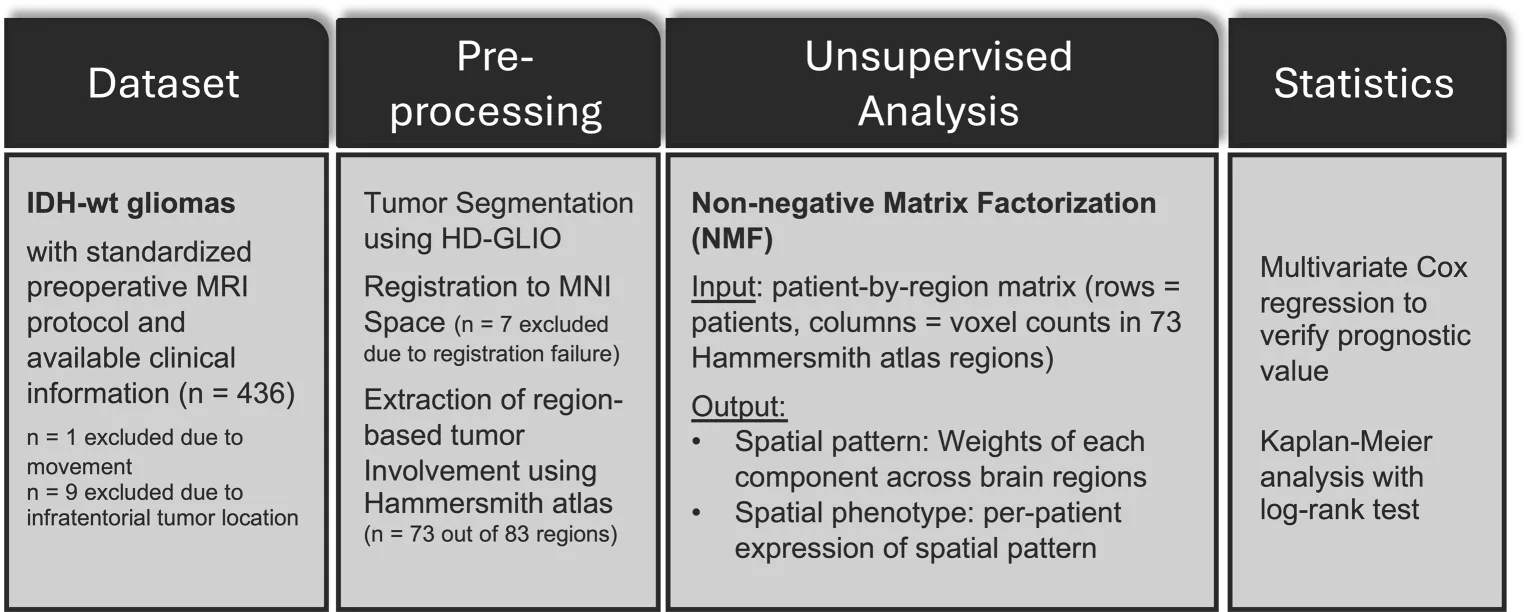
Study Workflow: Spatial Phenotyping of IDH-wt gliomas. Flowchart summarizing patient selection, imaging preprocessing, NMF-based spatial phenotyping using voxel counts across 73 brain regions, and survival analysis via Kaplan-Meier and Cox regression

Out of 446 patients diagnosed with IDH-wt glioma, as classified by the WHO 2021 criteria, 445 patients had imaging of sufficient quality, specifically, high-resolution T1-weighted images, to allow spatial normalization and anatomical mapping. The final dataset contained 429 patients (Fig. 1 depicts the workflow with the excluded patients, 9 patients due to infratentorial tumor location, and 7 patients due to registration failure). Demographics and clinical characteristics can be found in Table 1.

**Table 1:** Patient Demographics and Clinical Characteristics of the Total Cohort and the High-Confidence Subset Used for Dominant Pattern Analysis. P‑values reflect comparisons between the high-confidence subset and the remaining patients. Categorical variables were compared using the Chi-squared test; continuous variables using the independent t‑test; overall survival (OS) was compared using the log-rank test.

		Total Dataset	Dominant Phenotype Subset	*p*-value
*Patients*	*n*	429	171	–
*Sex:*				
Female	*n* (%)	240 (56%)	100 (58%)	0.39
*Age*				
< 70 years	*n* (%)	310 (72%)	130 (76%)	0.16
*ECOG status:*				
> 2	*n* (%)	16 (4%)	7 (4%)	0.75
*Extent of resection:*				
Biopsy	*n* (%)	89 (21%)	15 (9%)	< 0.001
Subtotal resection		229 (54%)	98 (59%)	
Total resection		103 (24%)	54 (32%)	
*OS survival (months)*	Median (95% CI)	16.8 (14.7–19.2)	20.0 (18.7–23.8)	0.002
*MGMT promotor:*				
Methylated	*n* (%)	185 (43%)	68 (40%)	0.25
*Tumor volume (cm*^*3*^*)*	Mean ± sd	84 ± 60	66 ± 52	< 0.001

### Unsupervised Analysis of Spatial Patterns Using NMF

#### Pretest

An initial NMF stability analysis identified six robust components, representing an interpretable set of spatial phenotypes that capture major variance in tumor distribution while minimizing overfitting (see Suppl. Fig. 1). A rank of six was selected as it represents a balance between stability and interpretability, with high cophenetic and silhouette values and only minor decreases in residuals beyond this point.

#### Description of Latent Spatial Pattern

**Fig. 2:**
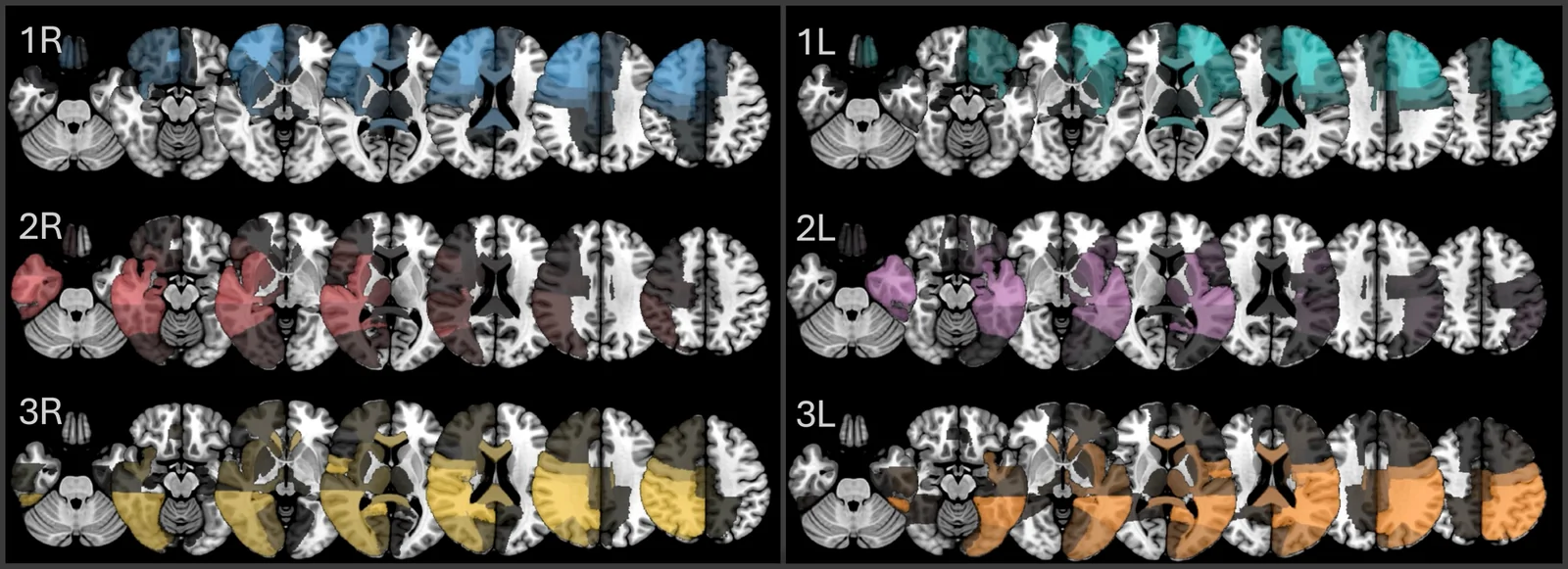
Spatial patterns derived from non-negative matrix factorization applied to voxel-based tumor distributions across 73 brain regions. Components are lateralized and labeled as 1L/1R (frontal–striatal), 2L/2R (temporal), and 3L/3R (parietal–occipital) patterns. Color hue identifies the corresponding spatial pattern, while color intensity reflects the degree of regional involvement—brighter tones indicate stronger contributions, and darker tones indicate minimal involvement within that pattern

The NMF analysis of IDH-wt gliomas revealed six distinct spatial patterns, each associated with a unique anatomical distribution of tumor involvement, including frontal–striatal (1L/1R), temporal (2L and 2R), and parietal–occipital regions (3L and 3R). Figure 2 presents a visual depiction of the components, and Table 2 shows a summary of the five areas with the highest weights per pattern. For a more detailed visualization, see Supplementary Fig. 2 with a depiction of the weights for all anatomical areas and spatial patterns. Interestingly, the patterns revealed a nearly symmetrical appearance for the hemispheres.

**Table 2:** Top-five areas for each spatial pattern are based on their weights per anatomical region.

Left Frontal-Striatal (1L)	Right Frontal-Striatal (1R)	Left Temporal (2L)	Right Temporal (2R)	Left Parietal-Occipital (3L)	Right Parietal-Occipital (3R)
Middle frontal gyrus	Middle frontal gyrus	Middle and inferior temporal gyrus	Middle and inferior temporal gyrus	Inferiolateral remainder parietal lobe	Superior parietal gyrus
Superior frontal gyrus	Superior frontal gyrus	Anterior temporal lobe (medial)	Superior temporal gyrus (posterior)	Superior parietal gyrus	Inferiolateral parietal lobe
Caudate nucleus	Precentral gyrus	Superior temporal gyrus (posterior)	Anterior temporal lobe (medial)	Postcentral gyrus	Postcentral gyrus
Inferior frontal gyrus	Caudate nucleus	Fusiform gyrus	Fusiform gyrus	Posterior temporal lobe	Posterior temporal lobe
Precentral gyrus	Inferior frontal gyrus	Superior temporal gyrus (anterior)	Superior temporal gyrus (anterior)	Lateral remainder occipital lobe	Lateral remainder occipital lobe

#### Description of Spatial Phenotypes

Each patient’s tumor could be expressed as a weighted combination of multiple spatial patterns reflecting the heterogeneous spatial invasion of the individual phenotype.

**Fig. 3:**
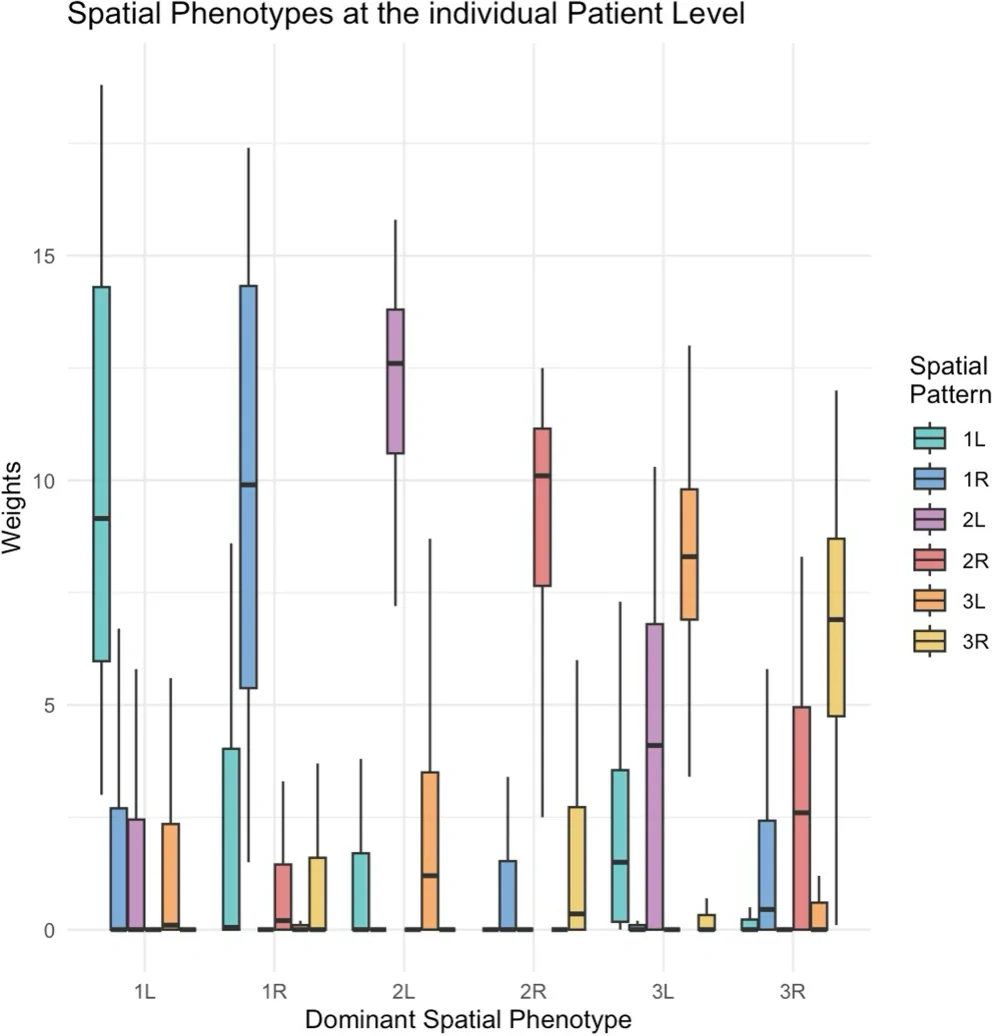
Distribution of Spatial Component Weights Across Dominant Phenotypes. Boxplots showing the distribution of individual topology weights across patients, grouped by their dominant spatial phenotype. Each box represents the expression of all spatial components within patients assigned to a given dominant pattern, highlighting co-expression and potential transitional or mixed phenotypes. Color indicates the assigned spatial pattern derived from the NMF-based decomposition of tumor location

Figure 3 aggregates the patient-level results by assigning the most dominant spatial phenotype (highest weights of the spatial pattern) and shows the distribution of component weights for the remaining spatial pattern. As expected, samples in each cluster display the highest weights for their corresponding dominant spatial pattern (e.g., cluster 1L shows the highest weights for pattern 1L), confirming the robustness of the clustering based on spatial location. Importantly, this decomposition also reveals a rich variability in spatial phenotypes across individual tumors. While dominant patterns characterize each cluster, many patients exhibit non-negligible weights for secondary patterns, indicating the presence of cross-pattern contributions. For example, patients in clusters 3L and 2L often show elevated weights from multiple spatial patterns, highlighting the existence of transitional phenotypes.

### Spatial Patterns as Predictors of Survival

The detailed results of the Cox proportional hazards models can be found in Table 3.

**Table 3:** Multivariable Cox regression models evaluating the prognostic value of spatial phenotypes (1L/R = frontal-striatal, 2L/R = temporal, 3L/R = parietal-occipital) alongside tumor volume and clinical covariates. Hazard ratios (HR), 95% confidence intervals (CI), and *p*-values are reported. C‑index values reflect model discrimination. Likelihood ratio test (LRT) statistics were performed to compare two nested Cox proportional hazards models with and without spatial phenotype terms.

Models	Parameter	HR, 95% CI, *p*-value	C index	LRT
*Model 1A*	Tumor volume	1.003 (1.001–1.005), 0.0177	0.5665	Χ^2^ = 34.697, df = 6, *p* < 0.0001
*Model 1B*	1L	1.111 (1.065–1.158), < 0.0001	0.6359	
	1R	1.101 (1.055–1.149), < 0.0001		
	2L	1.074 (1.030–1.120), 0.001		
	2R	1.064 (1.019–1.110), 0.005		
	3L	1.145 (1.086–1.207), < 0.0001		
	3R	1.164 (1.098–1.234), < 0.0001		
	Tumor volume	0.992 (0.987–0.997), 0.001		
*Model 2A*	Tumor volume	1.003 (1.001–1.006), 0.006	0.6373	Χ^2^ = 33.013, df = 6, *p* < 0.0001
	Age: < 70	0.595 (0.454–0.781), < 0.0001		
	Sex: female	1.063 (0.832–1.36), 0.625		
	MGMT: unmethylated	1.769 (1.37–2.284), < 0.0001		
*Model 2B*	1L	1.111 (1.065–1.158), < 0.0001	0.6777	
	1R	1.104 (1.058–1.152), < 0.0001		
	2L	1.065 (1.022–1.109), 0.003		
	2R	1.062 (1.017–1.108), 0.006		
	3L	1.145 (1.085–1.209), < 0.0001		
	3R	1.147 (1.08–1.218), < 0.0001		
	Tumor volume	0.993 (0.988–0.997), 0.002		
	Age: < 70	0.652 (0.489–0.869), 0.004		
	Sex: female	1.043 (0.812–1.341), 0.741		
	MGMT: unmethylated	1.839 (1.417–2.387), < 0.0001		
*Model 3A*	Tumor volume	1.003 (1.000–1.005), 0.052	0.6573	Χ^2^ = 29.031, df = 6, *p* < 0.0001
	Age: < 70	0.556 (0.421–0.733), < 0.0001		
	Sex: female	1.012 (0.784–1.305), 0.930		
	MGMT: unmethylated	1.972 (1.514–2.568), < 0.0001		
	Resection: subtotal	1.191 (0.796–1.782), 0.396		
	Resection: total	0.711 (0.459–1.104), 0.129		
	ECOG: > 2	2.187 (1.152–4.151), 0.017		
*Model 3B*	1L	1.113 (1.066–1.162), < 0.0001	0.6923	
	1R	1.092 (1.043–1.142), 0.0001		
	2L	1.076 (1.032–1.123), 0.001		
	2R	1.065 (1.020–1.113), 0.005		
	3L	1.139 (1.077–1.205), < 0.0001		
	3R	1.137 (1.069–1.210), < 0.0001		
	Tumor volume	0.992 (0.987–0.997), 0.001		
	Age: < 70	0.600 (0.449–0.802), 0.001		
	Sex: female	0.980 (0.755–1.272), 0.879		
	MGMT:unmethylated	2.095 (1.598–2.747), < 0.0001		
	Resection: subtotal	1.349 (0.896–2.033), 0.152		
	Resection: total	0.886 (0.556–1.412), 0.610		
	ECOG: > 2	2.095 (1.071–4.099), 0.031		

In the baseline model including tumor volume only (Model 1A), tumor volume was modestly associated with increased hazard (HR = 1.003, *p* *=* 0.02). When spatial pattern phenotypes were added (Model 1B), all six NMF-derived spatial components (1L, 1R, 2L, 2R, 3L, and 3R) were significantly associated with increased hazard, with the highest risks in 3L (HR = 1.145, *p* < 0.0001) and 3R (HR = 1.164, *p* < 0.0001). In this augmented model, the tumor volume effect reversed direction (HR = 0.992, *p* = 0.0006), consistent with shared prognostic information between global tumor burden and its spatial distribution.

In covariate-adjusted models (Models 2A–2B), MGMT unmethylated status was associated with an increased hazard (HR = 1.769 and 1.839, *p* < 0.0001), whereas younger age (< 70 years) was associated with decreased hazard (HR = 0.595 and 0.652, *p* < 0.0001), sex showed no significant effect. Addition of spatial phenotypes again resulted in significant hazard increases across all NMF components, accompanied by an inverse volume effect (HR = 0.993, *p* = 0.002).

Fully adjusted models including ECOG performance and resection status (Model 3) confirmed the independent prognostic value of all spatial phenotypes (HR range: 1.065–1.139, all *p* < 0.005). In these models ECOG > 2 and MGMT unmethylated status remained strong adverse factors, while extent of resection did not reach statistical significance.

Proportional hazards diagnostics showed no relevant violations for the NMF components across models, while tumor volume and age demonstrated mild time-dependence, reflected in borderline or significant Schoenfeld residual tests (Supplement Table 1).

Age-stratified sensitivity analyses yielded comparable effect sizes and significance levels for spatial phenotypes (Supplement Table 2), confirming the robustness of the findings. Assessment of multicollinearity revealed low variance inflation factors for all predictors (VIFs predominantly 1.2–2.5, maximum < 3.5), indicating that tumor volume and spatial phenotypes can be reasonably interpreted within the same model. Accordingly, the inversion of the tumor volume hazard ratio after inclusion of spatial phenotypes reflects adjustment for spatial tumor distribution rather than model instability.

Although multiple spatial phenotypes were tested, most associations were highly significant (typically *p* < 0.001) and would remain statistically significant under conservative multiple-comparison corrections. Therefore, interpretation is focused on effect size consistency and robustness across models rather than nominal *p*-values alone.

Across all model tiers, inclusion of spatial phenotypes significantly improved model fit compared with volume-only or covariate-only models (likelihood ratio tests *p* < 10⁻^5^) and increased concordance indices.

### Patient Classification Based on Dominant Spatial Phenotypes

To investigate the prognostic relevance of spatial tumor location, glioma patients were stratified into six dominant spatial phenotypes (1L, 1R, 2L, 2R, 3L, 3R) based on the most prominent pattern. To isolate patients with well-localized tumors, we applied a threshold to the NMF assignment probabilities (> 0.75), retaining only those whose tumors were dominated by a single spatial phenotype. The resulting subset comprised 171 patients (39.9% of the total cohort; Table 1, column: Dominant Phenotype Subset) distributed across the six spatial components as follows: 1L (*n* = 24), 1R (*n* = 27), 2L (*n* = 25), 2R (*n* = 34), 3L (*n* = 25), and 3R (*n* = 36).

As expected, patients in this subset had significantly smaller tumor volumes and a higher frequency of gross total resection, reflecting the exclusion of large or spatially diffuse tumors. These differences are consistent with the goal of mapping survival outcomes to distinct tumor locations while minimizing confounding from multifocal or anatomically ambiguous cases. Importantly, there were no significant differences in demographic characteristics or molecular markers, supporting the representativeness of the subset.

Hazard ratio estimates indicated that patients characterized by a dominant pattern in the left parieto-occipital region (3L) had an increased risk of death compared to the average hazard across all clusters (HR = 1.98, 95% CI: 1.13–3.46, *p* = 0.008). No other clusters showed statistically significant deviations from the average hazard.

**Fig. 4:**
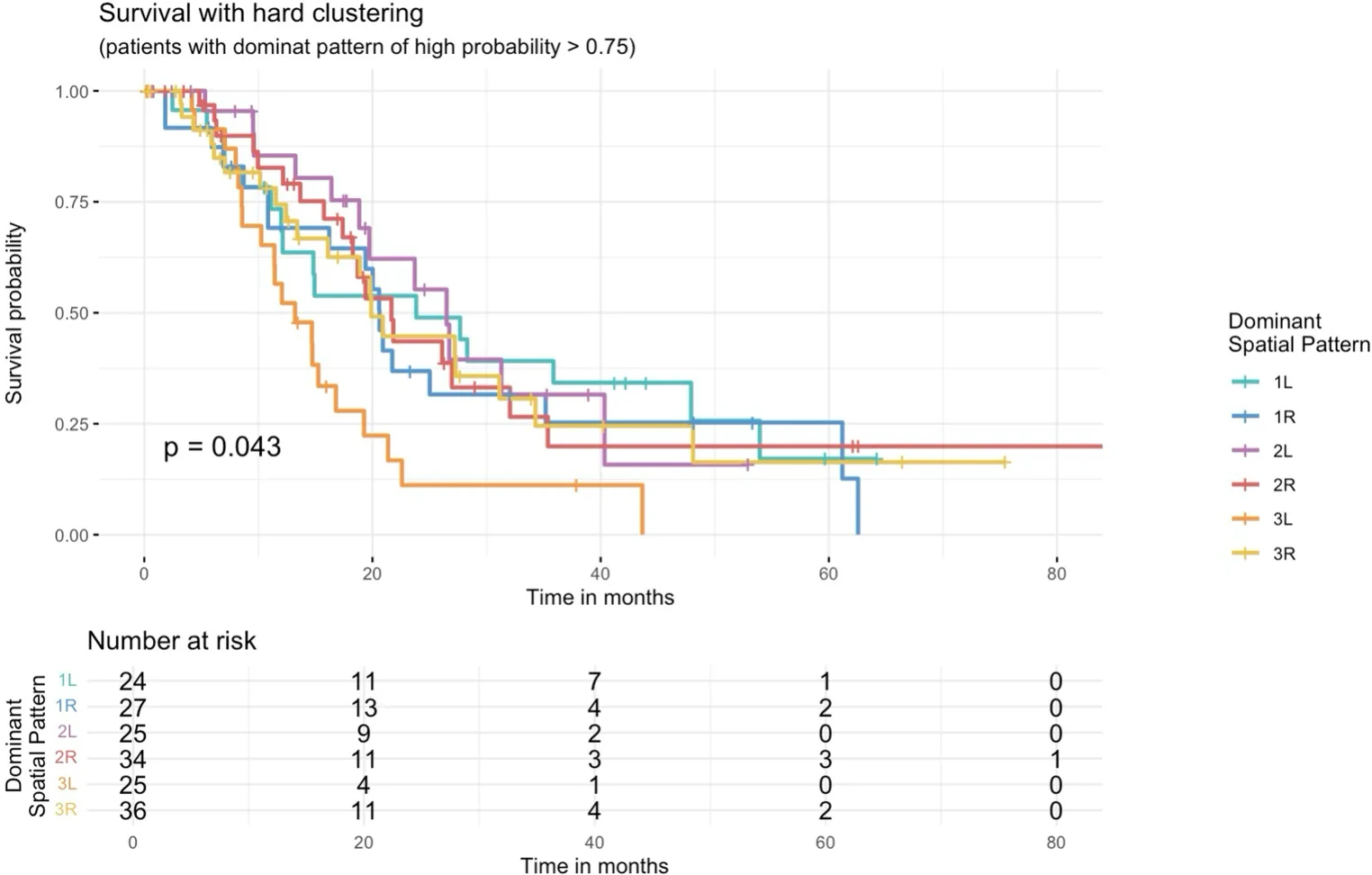
Kaplan-Meier diagram for hard clustering using a subset based on dominant weight with a probability > 0.75

Kaplan–Meier survival analysis revealed overall differences in survival across spatial clusters (log-rank test, *p* = 0.043; Fig. 4). Specifically, patients assigned to the 3L phenotype exhibited substantially reduced survival probabilities with a median survival of 13.2 months (10.3–21.4 months) relative to other clusters (median survival between 19.9 and 26.5 months). Unadjusted pairwise log-rank comparisons showed poorer survival for the 3L cluster compared to 1L, 1R, 2L, 2R, and 3R (uncorr. *p* for the comparisons: 0.028, 0.038, 0.005, 0.010, 0.031).

## Discussion

This study used NMF to uncover six distinct latent spatial patterns of tumor involvement in IDH-wt gliomas. These spatial phenotypes, distributed across symmetrical left and right hemispheric regions, not only captured the heterogeneity of tumor invasion but also exhibited strong prognostic value.

Our analysis showed that while tumors could be represented as combinations of multiple spatial patterns, a dominant phenotype typically emerged for each patient. Notably, tumors predominantly located in the left parieto-occipital region (3L) were associated with significantly reduced survival, with hazard ratios that remained robust across multiple Cox regression models adjusting for tumor volume, molecular status, clinical covariates, and performance metrics. These findings suggest that the spatial location of tumor burden, as captured by NMF-derived phenotypes, holds independent prognostic significance beyond established clinical and molecular markers.

Our results align with prior evidence that anatomical location is a critical determinant of glioma prognosis. Tumors involving deep or eloquent regions are known to confer worse survival, in part due to surgical inaccessibility and more aggressive biological profiles [22, 23]. Consistent with studies reporting shorter survival in central and occipital tumors [12, 24], the parieto-occipital pattern (3L) was most strongly associated with poor outcomes. This spatial pattern overlaps with posterior association cortices, including the precuneus and angular gyrus, regions involved in the default mode network and visuospatial integration. Infiltration of these areas may disrupt higher-order cognitive and integrative functions, potentially contributing to the elevated hazard observed in this group [25, 26]. Furthermore, the involvement of major white matter tracts, such as the corticospinal tract and inferior fronto-occipital fasciculus, may limit surgical resectability and thus compound the prognostic impact [27].

Interestingly, while earlier reports highlighted the temporal lobe—especially the left hemisphere—as a site of adverse prognosis [9], our findings indicate that temporal components, particularly on the right, were among the least associated with mortality. This is consistent with more recent analyses suggesting improved survival in temporal gliomas [12, 28], potentially reflecting improved surgical accessibility and higher rates of complete resection in this region [29].

We build upon the meta-topological framework introduced by Kernbach et al. [21], which applied NMF to categorize neuroepithelial tumors according to their spatial architecture. Unlike that study, which examined a heterogeneous mixture of glioma subtypes, our analysis was restricted to IDH-wt gliomas. This focused cohort allowed us to preserve spatial features as continuous variables rather than imposing categorical assignments, thereby enabling the identification of mixed or transitional spatial phenotypes in tumors with substantial weights across multiple spatial components. Such granularity provided finer prognostic resolution and revealed spatial gradients that likely mirror underlying neurodevelopmental and connectivity-related vulnerabilities [30, 31]. Furthermore, by integrating clinical variables known to improve survival modeling [10, 32], we demonstrate the added value of spatial phenotyping in a translational context.

Several limitations should be acknowledged. First, the retrospective and single-institution nature of our dataset may limit generalizability. Although our cohort is large and well-characterized, external validation is necessary to confirm the reproducibility of spatial phenotypes across institutions, scanners, and segmentation pipelines. Second, although we used a validated segmentation and registration pipeline, errors in preprocessing (e.g., partial volume effects, misregistration) and our single reviewer approach may affect the accuracy of regional voxel counts.

The choice of the dominant phenotype threshold represents a compromise between phenotypic specificity and statistical power; alternative thresholds resulted in either reduced purity or insufficient sample sizes for robust modeling. Finally, our cross-sectional analysis does not account for temporal dynamics such as tumor progression or treatment response. Future work could extend this framework to longitudinal imaging data to study how spatial phenotypes evolve over time and with therapy.

Despite these limitations, our findings support the hypothesis that anatomical tumor distribution contains a latent structure that influences survival and is, therefore, of clinical relevance. By extracting and modeling these patterns using NMF, we provide evidence that spatial phenotypes are independently associated with survival in IDH-wt glioma and offer a novel axis of stratification that complements molecular and volumetric markers. Future studies should aim to integrate spatial phenotypes with radiogenomic and transcriptomic data to elucidate the biological basis of these patterns and explore their potential role in guiding therapy.

In conclusion, this study introduces a robust framework for spatial phenotyping of gliomas using standard imaging data and demonstrates its prognostic relevance in a large cohort of IDH-wt tumors. Our results indicate that tumor topography serves as an imaging-based biomarker, reflecting underlying biological gradients with significant implications for prognosis and therapeutic decision-making. As neuro-oncology moves toward increasingly personalized approaches, spatial phenotyping may offer a new lens through which to understand and manage the heterogeneity of gliomas.

## Methods

### Dataset

The retrospective study was approved by the local ethics committee with an exemption from the requirement of informed consent (S-784 2018). We included 446 consecutive patients diagnosed with IDH-wt glioma per WHO CNS5 criteria [1] between 2009 and 2020, all of whom were confirmed to be 1p/19q-codeletion negative and underwent preoperative MRI. IDH mutation status and O6-methylguanine-DNA methyltransferase (MGMT) status were determined via DNA methylation profiling [33]. Survival data were obtained from medical records, with right-censoring applied when necessary.

High-resolution T1-weighted images were acquired using 3D MPRAGE sequences on 3 T Siemens scanners (Skyra, Verio, TrioTim, and Prisma; software versions syngo MR B17–E11) with the following parameters: TR = 1.71–1.96 s, TE = 3.25–4.04 ms, TI = 0.8–1.1 s, flip angle = 15°, voxel size = 0.8–1.3 mm isotropic, matrix size = 290–512 in-plane, GRAPPA acceleration factor = 2, partial Fourier = 0.75–1.0, using 16–32 channel head/neck coils. Imaging protocols followed international clinical standards and included T1-weighted sequences before and after intravenous administration of gadoterate meglumine (0.1 mmol/kg, Dotarem; Guerbet), as well as axial 2D FLAIR and T2-weighted sequences, in line with the current consensus recommendations [34].

### Imaging Analysis and Preprocessing

Tumor segmentation was performed using a modified nnUNet version of the HD-GLIO application [35, 36], enabling automated identification of necrotic, contrast-enhancing, and non-enhancing T2-weighted/FLAIR hyperintense tumor components. HD-GLIO is trained on brain tumor data and operates on non–brain-extracted images, thereby avoiding removal of superficial tumor components. Individual tumor components were merged to form a unified mask per subject and manually reviewed by MF, a former neuroradiology resident with six years of experience. The resulting tumor masks were subsequently used within the *fsl_anat* function from the FMRIB Software Library (FSL) toolbox to account for tumor tissue during preprocessing.

The function enabled the registration to 1 mm MNI space (https://fsl.fmrib.ox.ac.uk/fsl/fslwiki/Atlases) [37]using both linear and nonlinear transformations. Spatially normalized tumor masks were mapped to the Hammersmith brain atlas, which subdivides the brain into 83 regions [38, 39]. To ensure anatomical relevance for predominantly supratentorial tumors and to avoid confounding by infratentorial structures and CSF spaces, ten regions were excluded from further analyses: brainstem; left and right cerebellum; left and right lateral ventricles (temporal horn and non–temporal horn); third ventricle; and left and right substantia nigra. Nine patients with infratentorial tumors were removed, resulting in a final cohort of 429 patients across 73 regions.

### Unsupervised Analysis of Spatial Patterns Using NMF

The following data analysis steps were performed in R, version 4.2.1. To uncover latent spatial topographies of glioma distribution, we applied non-negative matrix factorization (NMF) using the NMF package [40], implementing the multiplicative update algorithm by Lee and Seung [41].

NMF is an unsupervised machine learning technique that decomposes high-dimensional non-negative data into a set of interpretable components and corresponding coefficients. In this context, each component represents a recurrent spatial pattern of tumor involvement—referred to here as spatial phenotypes—while each patient is represented as a weighted combination of these patterns. This decomposition revealed interpretable, topographic signatures that captured the heterogeneity of glioma spread across patients in a continuous and anatomically structured manner.

For each patient, voxel counts were computed per anatomical region, producing a structured spatial matrix of tumor distribution. Log_2_ transformation (with pseudocount 1) was applied to reduce skew and improve NMF performance.

To determine the optimal number of components (k), we applied NMF to the full dataset of all patients. Values from 2 to 15 were tested across 50 runs each. Final model selection was based on interpretability, consensus stability, and standard quality metrics. To assess robustness, we performed 200 additional NMF runs with random initializations using the full patient cohort, which confirmed the stability of the six-component solution.

No separate hold-out dataset was used, as NMF is an unsupervised method; however, consensus clustering and repeated initialization provided internal validation of the spatial patterns.

### Survival Analysis

Multivariate Cox proportional hazards regression was performed to assess the prognostic value of spatial phenotypes. NMF component weights were entered into Cox models on their native scale without additional normalization or standardization, in order to preserve direct correspondence with the original spatial tumor load decomposition. Models adjusted for age (binarized as < 70 vs. ≥ 70 years), sex, tumor volume, MGMT status, extent of resection, and Eastern Cooperative Oncology Group (ECOG) status. Comparisons between baseline and phenotype-augmented models were evaluated via likelihood ratio tests.

The proportional hazards (PH) assumption was evaluated using Schoenfeld residuals for all covariates. Where mild deviations were observed, sensitivity analyses using age-stratified Cox models were performed to confirm the robustness of spatial phenotype effects. Multicollinearity between tumor volume and NMF components was assessed using variance inflation factors (VIFs) derived from the model design matrix. Likelihood ratio tests were used to compare baseline models with phenotype-augmented models. Model discrimination was quantified using the concordance index.

### Patient Classification Based on Dominant Spatial Phenotypes

For downstream analyses, each patient was assigned to the spatial phenotype with the highest weight, implementing a hard clustering strategy. To minimize volume-related effects and avoid inclusion of transitional phenotypes with substantial contributions from multiple spatial patterns, a dominant pattern probability of 0.75 was selected to balance phenotypic purity with sufficient sample size for survival analyses. This filtering step ensured that the analysis focused on anatomically distinct and spatially well-defined tumor locations.

Estimated marginal means (EMMs) on the hazard ratio (HR) scale [42] and pairwise contrasts were computed to evaluate whether hazard ratios for individual clusters significantly differed from the overall average hazard. Kaplan–Meier survival analysis was conducted for the different phenotypes.

## Supplementary Information

ESM1: Supplementary material 1

## Data Availability

Due to institutional and legal restrictions, patient-level clinical, survival, pathological, and imaging data cannot be shared with third parties. De-identified, derived region-wise tumor load matrices are available from the corresponding author upon reasonable request. All analysis code used in this study will be made publicly available via a GitHub repository. Derived data supporting the findings of this study are included in the article and its supplementary materials.
