# Supplementary material for: From Anatomy to Outcome: Linking Glioma Location Patterns to Survival Using Non-Negative Matrix Factorization
Source: Clin Neuroradiol. 2026 Mar 27;36(3):921–30. doi: 10.1007/s00062-026-01642-8 (PMC13521997; doi:10.1007/s00062-026-01642-8)
Supplement: Supplementary file 1 [file 62_2026_1642_MOESM1_ESM.docx]

Supplementary Information file

From Anatomy to Outcome:

Linking Glioma Location Patterns to Survival using Non-negative Matrix Factorization

***Supplement Figure 1.*** NMF pretest for determination of cluster number. The cophenetic coefficient and dispersion suggest avoiding rank 4 (instability spike). The silhouette score shows better clustering quality around rank 6. The explained variance and residual decrease start to plateau after 6–8 components. Overall, rank 6 strikes a good balance.

***Supplement Figure 2.*** Regional Weights by Spatial Pattern: Left–Right Comparison. Bar plot showing the absolute weights for left–right differences across anatomical regions, stratified by spatial pattern. Weights are displayed per region and faceted to illustrate variation within each broader anatomical grouping. Color indicates the spatial pattern derived from NMF components. *Corpus callosum was displayed as absolute weight.

**Supplement Table 1:** Proportional hazards diagnostics and multicollinearity of Cox regression models including spatial tumor phenotypes (NMF components)

| **Model** | **Covariate** | **Schoenfeld residual χ²** | **df** | **p-value (PH test)** | **VIF** | **count** | **nb** |
| --- | --- | --- | --- | --- | --- | --- | --- |
| **Model 1B** | Tumor volume | 6.43 | 1 | 0.011 | 3.37 | 1 | 1 |
|  | 1L | 3.82 | 1 | 0.051 | 2.04 | 5 | 2 |
|  | 1R | 1.73 | 1 | 0.189 | 2.24 | 6 | 3 |
|  | 2L | 1.39 | 1 | 0.238 | 2.33 | 7 | 4 |
|  | 2R | 0.17 | 1 | 0.68 | 1.95 | 8 | 5 |
|  | 3R | 0.5 | 1 | 0.481 | 2.13 | 9 | 6 |
|  | 3L | 1.17 | 1 | 0.279 | 2.46 | 10 | 7 |
| **GLOBAL test** | – | 14.04 | 7 | 0.05 |  |  |  |
| **Model 2B** | Tumor volume | 4.36 | 1 | 0.037 | 3.45 | 1 | 8 |
|  | Age < 70 | 10.8 | 1 | 0.001 | 1.06 | 2 | 9 |
|  | Sex | 0.34 | 1 | 0.56 | 1.01 | 3 | 10 |
|  | MGMT unmethylated | 0.0004 | 1 | 0.983 | 1.02 | 4 | 11 |
|  | 1L | 3.23 | 1 | 0.072 | 2.08 | 5 | 12 |
|  | 1R | 1.89 | 1 | 0.169 | 2.16 | 6 | 13 |
|  | 2L | 1.82 | 1 | 0.177 | 2.31 | 7 | 14 |
|  | 2R | 0.17 | 1 | 0.684 | 1.93 | 8 | 15 |
|  | 3R | 0.66 | 1 | 0.417 | 2.23 | 9 | 16 |
|  | 3L | 0.52 | 1 | 0.471 | 2.69 | 10 | 17 |
| **GLOBAL test** | – | 25 | 10 | 0.005 | - |  |  |
| **Model 3B** | Tumor volume | 5.33 | 1 | 0.021 | 1.36 | 1 | 18 |
|  | Age < 70 | 10.58 | 1 | 0.001 | 1.06 | 2 | 19 |
|  | Sex | 0.31 | 1 | 0.58 | 1.03 | 3 | 20 |
|  | MGMT unmethylated | 0.002 | 1 | 0.965 | 1.04 | 4 | 21 |
|  | Resection subtotal | 3.85 | 2 | 0.146 | 1.73 | 5 | 22 |
|  | Resection total | – | – | – | 1.72 | 6 | 23 |
|  | ECOG >2 | 1.88 | 1 | 0.171 | 1.05 | 7 | 24 |
|  | 1L | 3.32 | 1 | 0.069 | 1.47 | 8 | 25 |
|  | 1R | 3.07 | 1 | 0.08 | 1.37 | 9 | 26 |
|  | 2L | 1.21 | 1 | 0.272 | 1.47 | 10 | 27 |
|  | 2R | 0.36 | 1 | 0.546 | 1.28 | 11 | 28 |
|  | 3R | 0.71 | 1 | 0.4 | 1.3 | 12 | 29 |
|  | 3L | 0.74 | 1 | 0.39 | 1.39 | 13 | 30 |
| **GLOBAL test** | – | 28.64 | 13 | 0.007 | – |  |  |

**Supplement Table 2:** Age-stratified Cox regression including spatial phenotypes.

Cox model stratified by age (<70 vs. ≥70 years). Age was included as a stratification variable and therefore no hazard ratio is estimated for age. The global proportional hazards test was not significant in the stratified model (p=0.12), indicating improved adherence to the PH assumption compared with unstratified models. Effect sizes of spatial phenotypes were highly consistent with the primary (unstratified) analyses.

| **Parameter** | **HR, 95% CI, p-value** |
| --- | --- |
| **1L** | 1.109 (1.062-1.158), <0.0001 |
| **1R** | 1.092 (1.044-1.143), <0.0001 |
| **2L** | 1.076 (1.032-1.123), 0.001 |
| **2R** | 1.063 (1.018-1.11), 0.006 |
| **3L** | 1.144 (1.082-1.21), <0.0001 |
| **3R** | 1.136 (1.068-1.208), <0.0001 |
| **tumor volume** | 0.992 (0.987-0.997), 0.001 |
| **Sex: female** | 0.947 (0.729-1.231), 0.685 |
| **MGMT: unmethylated** | 2.088 (1.594-2.735), <0.0001 |
| **resection: subtotal** | 1.291 (0.856-1.948), 0.224 |
| **resection: total** | 0.859 (0.539-1.37), 0.523 |
| **ECOG: > 2** | 2.018 (1.029-3.959), 0.041 |
